# The Benefit of Optimal Dietary Lipid Level for Black Seabream *Acanthopagrus schlegelii* Juveniles under Low-Salinity Environment

**DOI:** 10.1155/2022/2222029

**Published:** 2022-10-11

**Authors:** Min Jin, Xuejiao Li, Yuedong Shen, Yangguang Bao, Bingqian Yang, Zhaoxun Wu, Lefei Jiao, Qicun Zhou

**Affiliations:** ^1^Laboratory of Fish Nutrition, School of Marine Sciences, Ningbo University, Ningbo 315211, China; ^2^Key Laboratory of Aquaculture Biotechnology Ministry of Education, Ningbo University, Ningbo 315211, China

## Abstract

The present study was aimed at evaluating the regulatory effects of dietary lipid levels on growth performance, osmoregulation, fatty acid composition, lipid metabolism, and physiological response in *Acanthopagrus schlegelii* under low salinity (5 psu). An 8-week feeding trial was conducted in juvenile *A. schlegelii* with an initial weight of 2.27 ± 0.05 g, and six isonitrogenous experimental diets were formulated with graded levels of lipid: 68.7 g/kg (D1), 111.7 g/kg (D2), 143.5 g/kg (D3), 188.9 g/kg (D4), 239.3 g/kg (D5), and 269.4 g/kg (D6), respectively. Results indicated that fish fed with diet containing 188.9 g/kg lipid significantly improved growth performance. Dietary D4 improved ion reabsorption and osmoregulation by increasing the concentrations of Na^+^, K^+^, and cortisol in serum and activities of Na^+^/K^+^-ATPase as well as expression levels of osmoregulation related to gene expression levels in the gill and intestine. The expression levels of long chain polyunsaturated fatty acid biosynthesis-related genes were dramatically upregulated when dietary lipid levels increased from 68.7 g/kg to 189.9 g/kg with levels of docosahexaenoic (DHA), eicosapentaenoic (EPA), and DHA/EPA ratio being highest in the D4 group. When fish fed dietary lipid levels from 68.7 g/kg to 188.9 g/kg, lipid homeostasis could be maintained by upregulating *sirt1* and *pparα* expression levels, whereas lipid accumulation was observed in dietary lipid levels of 239.3 g/kg and over. Fish fed with high dietary lipid levels resulted in physiological stress related to oxidative stress and endoplasmic reticulum stress. In conclusion, based on weight gain, the optimal dietary lipid requirement of juvenile *A. schlegelii* reared at low-salinity water is 196.0 g/kg. These findings indicate that the optimal dietary lipid level can improve growth performance, n-3 LC-PUFA accumulation, and osmoregulatory ability and maintain lipid homeostasis and normal physiological functions of juvenile *A. schlegelii.*

## 1. Introduction

Generally, osmoregulation is an energy-dependent process for aquatic animals, and the animal body needs to consume more energy to maintain the balance of ion concentration in response to salinity changes [1]. Evidence shows that dietary lipid supplementation could not only provide energy but also have a positive impact on fish adaptation to a low-salinity environment [2]. Lipid is characterized by a high productivity and high proportion in biofilms, acting on cell membrane components and thus affecting cell membrane fluidity, and, hence, plays a key role in fish to adapt to salinity changes [3–6]. In addition, lipid can provide energy through *β*-oxidation in mitochondria and lipid droplet autophagy [7]; for instance, as one of the lipid metabolites, fatty acid can be esterified inter cells and then transported to mitochondria for oxidation to obtain energy [8, 9]. Moreover, the rate of ions entering and leaving cells can be affected to enhance and improve the osmoregulatory ability, through changing the membrane surface area and regulating Na^+^/K^+^-ATPase (NKA) activity [10]. Previous studies suggested that the changes of lipid ratio in membrane led to increase of membrane surface area and related ion pumps, thus improving ion transport rate and osmotic pressure regulation ability [11–13]. Therefore, optimal dietary lipid level not only can promote growth performance of farmed fish but also can improve the ability of fish to adapt to salinity changes; in turn, excessive dietary lipid intake could increase the burden on the fish body and reduce its ability to adapt to environmental changes [14]; hence, there is an urgent need to understand the optimal dietary lipid requirement for aquatic animals in different environments, for instance, low-salinity environment.

Lipids have the highest energy value among dietary energy sources, the effective utilization of energy of diet is the key to promote fish growth and reduce cost, and the utilization of dietary lipid can minimize organic pollution and eutrophication of water body to the maximum extent compared with protein and carbohydrate [15]. There have been a lot of studies on optimal dietary lipid requirement among various marine fish reared at normal water salinity, such as *Rachycentron canadum* (50 g/kg) [16], *Atractoscion nobilis* (150-180 g/kg) [17], *Sciaenops ocellatus* (7-14 g/kg), *Dicentrarchus labrax* (120 g/kg) [18], and *Acanthopagrus schlegelii* (83-136 g/kg) [19, 20]. A previous study demonstrated that the optimal dietary lipid levels of white shrimp (*Litopenaeus vannamei*) are various in different water salinity [21]. However, there is almost no research on the requirement of dietary lipid level of marine fish under low-salinity environment.

Many previous studies have reported that *A. schlegelii* has strong disease resistance and has the ability to tolerate a wide range of environmental conditions, which is becoming a very popular and commercially important marine fish species cultured in China, Japan, Korea, and other countries in Southeast Asia [22–25]. Seabream production in China reached almost 123,000 tons in 2020 [26]. Moreover, as a euryhaline fish, *A. schlegelii* is widely farmed under both normal/ambient salinity in seawater cages and low-salinity conditions in pond culture in China [23]. So it could be a good model to study the effects of low-salinity environment on physiological and biochemical responses of marine fish [22, 27]. Until now, the nutritional requirement of *A. schlegelii* under low-salinity environment remains unknown. Therefore, the present study was aimed at determining the optimal dietary lipid levels, exploring the regulatory effects of different dietary lipid levels on growth performance, osmoregulation, lipid metabolism, and physiological response of juvenile *A. schlegelii*, and providing a theoretical basis for marine fish reared under low-salinity environment.

## 2. Materials and Methods

### 2.1. Preparation of Experimental Diets

Six isonitrogenous (~40% crude protein) experiment diets with different levels of lipid were formulated, 68.7 g/kg (D1), 111.7 g/kg (D2), 143.5 g/kg (D3), 188.9 g/kg (D4), 239.3 g/kg (D5), and 269.4 g/kg (D6), respectively. Fish meal, soybean protein concentrate, and soybean meal were used as protein sources, and constant proportional fish oil and soybean oil were used as the main lipid sources. All ingredients were purchased from Ningbo Tech-Bank Feed Co. Ltd., Ningbo, China. Formula and nutrient composition of experimental diets are shown in Table 1, and the fatty acid compositions of experimental diets are presented in Table 2. The experimental diets were formulated according to the method used in our previous study [28] with a minor modification. Briefly, all dry ingredients were ground into fine powder with particle size < 80 *μ*m, and microcomponents such as minerals and vitamin premix were added followed by lipid and distilled water (35%, *w*/*w*). The ground ingredients were mixed in a Hobart type mixer and cold-extruded pellets produced (F-26, Machine factory of South China University of Technology) with pellet strands cut into uniform sizes (2 mm and 4 mm diameter pellets were prepared) (G-250, Machine factory of South China University of Technology). Pellets were exsiccated at 45°C by a blast dryer for 8 hours and to approximately 10% moisture, sealed in vacuum-packed bags, and stored at -20°C until used in the feeding trial.

### 2.2. Feeding Trial

*A. schlegelii* juveniles were obtained from a local commercial hatchery in Xiangshan Bay (salinity ranges from 23 to 25 psu in summer), Ningbo, China, and the feeding trial was carried out in Meishan Campus of Ningbo University (Ningbo, China). Prior to conduct the feeding trial, fish were cultured under 350 L (PE material, diameter of 1 m, and height of 1 m) recirculation aquaculture system (RAS) (HISHING Smart Equipment Co. Ltd., Qingdao, China) for two weeks to acclimate the environment. Water was purified with a filtration treatment of microfiltration machine and aerated by root blower aerator. During this period, fish were temporary rearing at low salinity (~5 psu) fed with a commercial diet (420 g/kg dietary protein, 120 g/kg crude lipid, Ningbo Tech-Bank Corp.). The low-salinity water was obtained by diluting natural seawater (~23 psu) with freshwater. *A. schlegelii* juveniles were acclimated from 23 psu to 5 psu salinity by decreasing 2 psu/day over approximately 9 days. A total of 540 juveniles with an initial weight of 2.27 ± 0.05 g were allocated to 18 tanks (350 L). Briefly, during water change, the amount of seawater and fresh water entering the system is controlled according to the monitored salinity. Fish were hand-fed to apparent satiation twice daily at 08:00 and 18:00 for eight weeks. During the experimental period, water conditions including temperature (25.5-26.8°C), salinity (4.86-5.17 psu), dissolved oxygen (9.24–9.89 mg/L), and pH (7.3-8.2 mg/L) were measured with YSI ProPlus (YSI, Yellow Springs, Ohio, USA).

### 2.3. Sample Collection

At the end of feeding trial, fish were anesthetized with tricaine methane sulphonate (MS-222) at 100 mg/L (Sinopharm Chemical Reagent Co., Ltd.). All fish in each tank were individually weighed and counted to determine weight gain (WG), specific growth rate (SGR), feed efficiency (FE), and survival rate (SR). Three fish per treatment (*n* = 3) were used to determine morphological parameters including viscerosomatic index (VSI), hepatosomatic index (HSI), and intraperitoneal fat ratio (IPF). Liver samples were also collected and stored at -80°C until further analysis of gene expression (pools of 3 fish per tank, *n* = 3), enzyme activity (pools of 3 fish per tank, *n* = 3), and fatty acid compositions (pools of 5 fish per tank, *n* = 3). Gill (cut along the gill arch) and intestine samples were collected and stored at -80°C until further analysis of gene expression and enzyme activity (pools of 3 fish per tank, *n* = 3). Kidney samples were collected for enzyme activity assay (pools of 3 fish per tank, *n* = 3). Blood samples were taken from the caudal vasculature of 10 fish per cage using 1.5 mL syringes and stored at 4°C for 12 h. Then, the blood samples were centrifuged at 956 × *g* for 10 min at 4°C to separate the serum for serum biochemical indices and enzyme activity analysis. Fresh liver tissues were collected into Bouin's fluid (Solarbio Life Sciences, Beijing, China) until further production of paraffin section. Samples were simply cleaned in 0.1 M PBS phosphate buffer (PBS, Beijing Solarbio Science & Technology Co. Ltd., Beijing, China), then quickly put into liquid nitrogen, and transferred to -80°C refrigerator for storage.

### 2.4. Proximate Composition Analysis

Crude protein, lipid, moisture, and ash contents in diets as well as lipid content in the liver were determined according to the methods of Association of Official Analytical Chemists [29]. Briefly, moisture was determined by drying the samples to a constant weight at 105°C. Protein was determined via the Dumas combustion method with a protein analyzer (FP-528, Leco, USA). Lipid was determined by the ether extraction method using a Soxtec System HT (Soxtec System HT6, Tecator, Sweden). Ash was determined using a muffle furnace run at 550°C for 8 h.

### 2.5. Assay of Osmoregulation Indices

The gill, kidney, and intestine samples were homogenized in 9 times volume (*w*/*v*) of precooled normal saline (0.89%) and then centrifuged at 4°C for 10 min at 956 × *g*. Cortisol in serum was determined with an ELISA kit (Shanghai Qiaodu Bioengineering Institute, Shanghai, China). In addition, the concentrations of sodium (Na^+^), potassium (K^+^), chloride (Cl^−^), and Na^+^/K^+^-ATPase (NKA) in both serum and tissue homogenates (intestine, kidney, and gill) were determined according to the manufacturer's instructions using commercial kits (Nanjing Jiancheng Bioengineering Institute, Nanjing, China). All the indices were measured via Multiskan microplate spectrophotometer (Thermo, USA). Briefly, the serum/tissue homogenate concentrations and determination wavelength in each index assay were as follows: Na^+^ (5%, 620 nm), K^+^ (10%, 450 nm), Cl^−^ (10%, 480 nm), and NKA (10%, 636 nm).

### 2.6. Fatty Acid Absolute Quantitative Assays

The absolute quantification of fatty acids in feed and liver samples was performed as follows: 1 mL methyl tricosanoate solution (23 : 0; 1 mg mL^−1^) internal standard was added to 12 mL glass screwed tubes and the solution reduced to dryness at room temperature (25°C) using a termovap sample concentrator (MIULAB NDK200-1 N, Hangzhou, China). After that, freeze-dried samples (approximately 0.2 g feed and 0.1 g liver) were added to the above-mentioned tubes before 3 mL transmethylation solution (99 mL methanol: 1 mL H_2_SO_4_: 0.025 g butylated hydroxytoluene) was added. After shaking for 10 min, the samples were incubated in a water bath at 80°C for 4 h. After cooling at room temperature, 1 mL n-hexane was added to the above mixture and shaken vigorously for 1 min, 1 mL ultrapure water added to promote layer separation, and the supernatant collected and filtered through a 0.22 *μ*m ultrafiltration membrane (Millipore, MA, USA) into a clean ampoule. The fatty acid methyl ester (FAME) solution in the ampoule was concentrated under nitrogen gas flow in a termovap sample concentrator, and the FAME was resuspended in 500 *μ*L n-hexane and stored at -20°C until analysis by gas chromatography-mass spectrometry (GC-MS; Agilent 7890B-5977A, Agilent Technologies, CA, USA). Absolute quantification of fatty acids was calculated according to the ratio of FAME peak areas to the internal standard (23 : 0) peak area, and results are presented as absolute concentration (mg g^−1^ feed or liver, dry matter).

### 2.7. Oxidative and Antioxidant Parameter Assays

The liver samples were homogenized in nine volumes (*w*/*v*) of ice-cold physiological saline 0.89% (*w*/*v*) and then centrifuged at 956 g for 10 min at 4°C. The supernatant was collected for detecting oxidative and antioxidant parameters. The concentrations of malondialdehyde (MDA), as well as enzymatic activities of superoxide dismutase (SOD), catalase (CAT), and total antioxidant capacity (T-AOC) in serum and tissue homogenates, were determined using assay kits according to the manufacturer's instructions (Nanjing Jiancheng Bioengineering Institute) and measured by Multiskan microplate spectrophotometer (Thermo, USA). Specifically, the liver homogenate concentrations and determination wavelength in each index assay were as follows: protein (1%, was diluted 10-fold), MDA (2.5%, 532 nm), CAT (20%, 405 nm), and T-AOC (10%, 520 nm). Prior calculations, the content of protein was adjusted according to different concentrations of liver homogenate.

### 2.8. Histological Analysis of the Liver

Fresh liver tissues were fixed in Bouin's fluid and sent to the company (Multisciences (Lianke) Biotech, Co., Ltd.) for paraffin section preparation. Briefly, dissect tissue as fast as possible, and then, immerse in fixative (Bouin's fluid) immediately. Trim tissue sample appropriately in chemical hood after fixation (at least 24 hours). Dehydrate in ethanol with incremental concentration from 75% to 100%. Then, they were embedded in paraffin and sliced into sections at 4 *μ*m using microtome. They were stained with hematoxylin and eosin (H&E), and images were acquired under a microscope (Nikon Eclipse CI, Tokyo, Japan). Additionally, the percentage of lipid vacuoles (PLV) in hepatic section was measured by Image Pro Plus 6.0 software (Media Cybernetics, Inc., Bethesda, MD, USA).

### 2.9. Total RNA Extraction, Reverse Transcription, and Real-Time PCR

Gene expression was determined by reverse-transcriptase quantitative PCR (qPCR), which was carried according to a method described in detail previously [30]. Briefly, total RNA was extracted from the target tissues of juvenile *A. schlegelii* using TRIzol reagent according to the manufacturer's instructions (Vazyme, China). Quantity and quality of isolated RNA were determined spectrophotometrically (NanoDrop 2000, Thermo Fisher Scientific, USA) and by 1.2% denaturing agarose gel electrophoresis, respectively. The cDNA was prepared from 1000 ng of DNAse-treated RNA and synthesized using PrimeScript™ RT Reagent Kit with gDNA Eraser (Perfect Real Time) (Vazyme). The housekeeping gene *β-actin* was used as reference gene after confirming its stability across the experimental treatments. The specific primers used for RT-qPCR were designed based on cDNA sequences of the corresponding genes in the NCBI database using Primer Premier 5.0 software (Table 3). Amplification was performed using a quantitative thermal cycler (LightCycler 96, Roche, Switzerland). The qPCR assays were performed in a total volume of 20 *μ*L, containing 1.0 *μ*L of each primer, 10 *μ*L of 2× conc. SYBR Green I Master (Vazyme), 2 *μ*L of 1/16 diluted cDNA, and 6 *μ*L DEPC-water. The thermal cycling conditions used for qPCR were as follows: 95°C for 2 min, followed by 45 cycles of 95°C for 10 s, 58°C for 10 s, and 72°C for 20 s. Calibration curves were prepared from six individual dilution concentration gradients of cDNA samples. Standard curves were generated using six different dilutions (in triplicate) of the cDNA samples, and the amplification efficiency was analyzed using the equation *E* = 10(–1/Slope) − 1. The amplification efficiencies of all genes were approximately equal and ranged from 89.1 to 102.8%. Gene expression data were presented as relative gene expression with regard to the expression of the ctrl group (reference group). The expression levels of the target genes were calculated using the 2^–*ΔΔ*Ct^ method as described previously [31].

### 2.10. Calculations and Statistical Analysis

The parameters were calculated as follows: Weight gain (WG, %) = 100 × ((final body weight–initial body weight)/initial body weight). Specific growth ratio (SGR, %day^−1^) = 100 × ((Ln final body weight (g) − Ln initial body weight) (g)/days). Feed efficiency (FE) = weight gain (g, wet weight)/feed consumed (g, dry weight). Survival (%) = 100 × (final fish number/initial fish number). Viscerosomatic index (VSI, %) = 100 × (visceral weight/wet body weight). Hepatosomatic index (HSI, %) = 100 × (liver weight/wet body weight). Intraperitoneal fat ratio (IPF, %) = 100 × (intraperitoneal fat weight/wet body weight).

Results are presented as means ± SEM (number of replicates as indicated). The relative gene expression results (qPCR analyses) were expressed as mean normalized ratios corresponding to the ratio between the copy numbers of the target genes and the copy numbers of the reference gene, *β-actin*. The expression levels of target gene were calculated relative to the expression level of *β-actin* and then presented as relative to the expression level in the D1 group. The homogeneity of variances (Levene's test) was checked prior ANOVA tests and then was analyzed by one-way analysis of variance (ANOVA) followed by Tukey's HSD test at a significance level of *P* ≤ 0.05. The statistical analyses were tested by SPSS software (IBM SPSS Statistics 20, USA).

The obtained hepatic fatty acid data were subjected to hierarchical cluster analysis (HCA) and principal component analysis (PCA) essentially as described previously with some minor modifications [34]. The data were normalized and integrated according to the Pareto scaling method before the processed data were subjected to supervised partial least squares discriminant analysis (PLS-DA) after importation into the SIMCA-P software (Version 11.0.0.0, Umetrics AB, Malmo, Sweden). The HCA was performed to analyze relationships between the different sample fatty acid profiles utilizing the Pearson correlation and average clustering algorithm subsequent to log2 transformation. Heat maps were used to visualize the complex fatty acid data sets organized as Pearson correlation matrices. The free online program Image GP (http://www.ehbio.com/ImageGP/index.php/) was used for both the HCA and heat map visualization.

## 3. Results

### 3.1. Growth Performance and Feed Utilization

**Figure 1:**
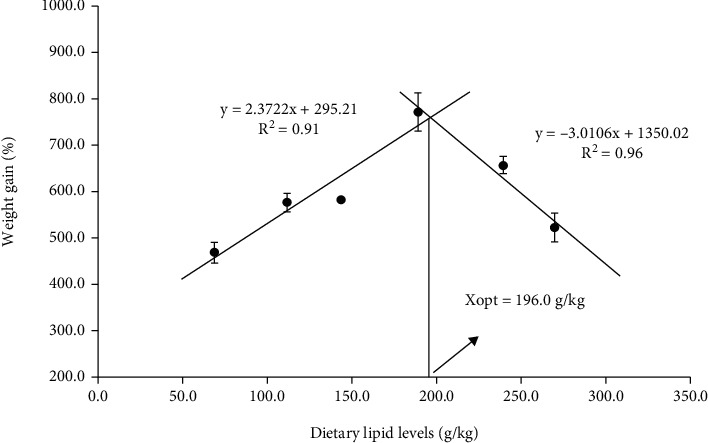
Relationship between weight gain (%) and dietary lipid levels based on linear broken-line model analysis, where Xopt represents the optimal dietary lipid level for the maximum weight gain of *Acanthopagrus schlegelii*. Each point represents the mean of three replicates per treatment.

The impacts of different dietary lipid levels on growth performance and feed utilization in juvenile *A. schlegelii* reared at low-salinity water are presented in Table 4. The highest values of WG, SGR, and FBW were recorded in D4 group and significantly higher than those in fish fed with the D1 and D6 diets (*P* < 0.05). Similar result was reflected in FE; the D4 treatment showed dramatically higher FE than that in fish fed with the dietary lipid levels below 188.9 g/kg (D1-D3) (*P* < 0.05). Based on WG, the optimal dietary lipid requirement of juvenile *A. schlegelii* under low-salinity environment is 196.0 g/kg by broken line model analysis (Figure 1). However, the SR did not show any statistical differences among dietary treatments (*P* > 0.05).

### 3.2. Key Indices of Osmoregulation

**Figure 2:**
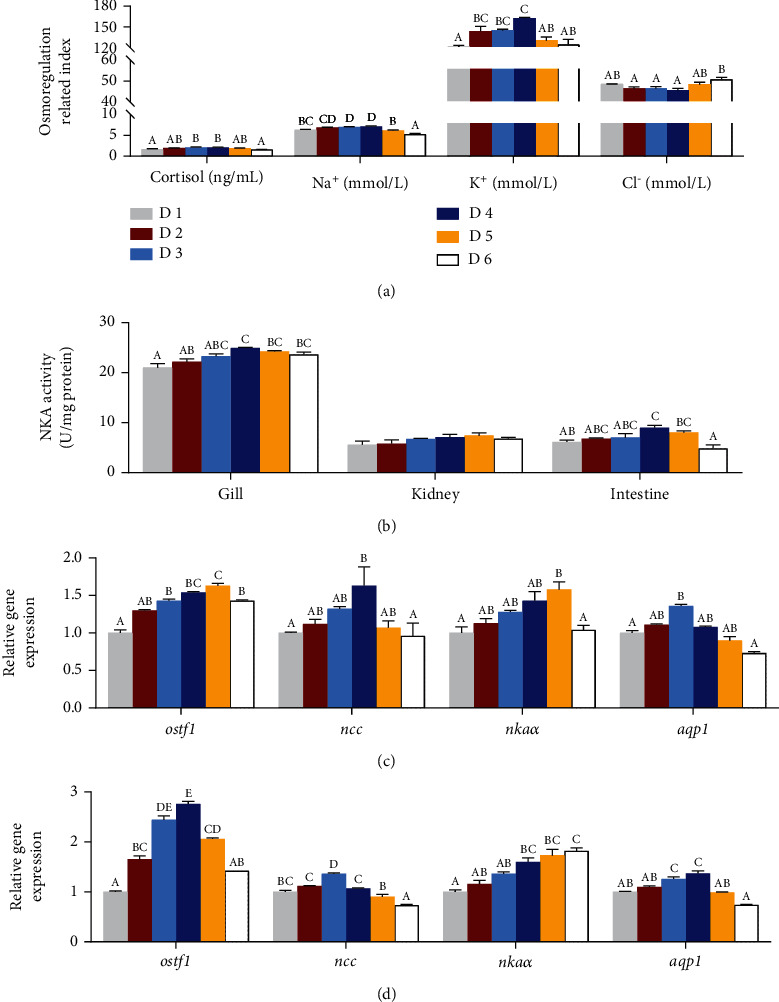
Effects of dietary lipid levels on osmoregulation ability of juvenile *Acanthopagrus schlegelii* reared at low-salinity water. Contents of serum cortisol and ions (a), NKA enzyme activity in the gill, kidney, and intestine (b), and expression levels of genes involved in osmoregulation in the gill (c) and intestine (d). The expression levels of target gene were calculated relative to the expression level of *β-actin* and then presented as relative to the expression level in the D1 group. Values are presented as means with standard errors represented by vertical bars (mean ± SEM, *n* = 3). Mean values for the same column with different superscript letters were significantly different (*P* < 0.05). *aqp1*: aquaporin 1; *ncc*: Na^+^-Cl^−^ cotransporter; *nkaα*: Na^+^/K^+^-ATPase; *ostf1*: osmotic stress transcription factor 1.

Dietary lipid levels had significant effects on osmoregulation related parameters of juvenile *A. schlegelii* under low-salinity environment (Figure 2). Significantly higher concentrations of serum cortisol, Na^+^, and K^+^ contents were recorded in fish fed the diet supplemented with 188.9 g/kg lipid (dietary D4) when compared to D1 and D6 groups (*P* < 0.05), with Cl^−^ changing in the opposite direction and showing lowest level in the D4 group (*P* < 0.05) (Figure 2(a)). The activity of NKA showed similar responses in different osmotic pressure regulating tissues (Figure 2(b)). Specifically, NKA activity in the gill and intestine increased with the increase of dietary lipid levels to reach a peak in the D4 group and then decreased (*P* < 0.05), while NKA activity in the kidney showed the same trend although no significant differences were found among treatments (*P* > 0.05). In the gill, the expression levels of osmotic stress transcription factor 1 (*ostf1*), sodium chloride transporter (*ncc*), aquaporin 1 (*aqp1*), and Na^+^/K^+^-ATPase *α* (*nkaα*) increased firstly and then decreased with the increase of dietary lipid levels (*P* < 0.05) (Figure 2(c)). The expression levels of *ostf1* and *nkaα* in the D5 group were significantly higher than fish fed with dietary D1 and D6 groups (*P* < 0.05). The significantly high expression levels of *ncc* and apq1 were recorded in D4 and D3 groups, respectively, and dramatically higher than the D1 and D6 groups (*P* < 0.05). Similar patterns were recorded for expression levels of *ostf1*, *ncc*, and *nkaα* in the intestine, which increased firstly and then decreased with the increase of dietary lipid levels, except for *nkaα* (*P* < 0.05) (Figure 2(d)). The expression levels of *ostf1* and *aqp1* in the D4 group were significantly higher than those in other groups (*P* < 0.05), except for D3 treatment. The highest expression level of *ncc* was found in the D3 group and markedly higher than that in other groups (*P* < 0.05). However, the gene expression level of *nkaa* was significantly increased with the increase of dietary lipid levels to reach a peak in the D6 group, which was significantly higher than that in D1, D2, and D3 groups (*P* < 0.05), but it had no statistical difference with the D4 and D5 treatments (*P* > 0.05) (Figure 2(d)).

### 3.3. Fatty Acid Composition and LC-PUFA Biosynthetic Pathway Markers in the Liver

**Figure 3:**
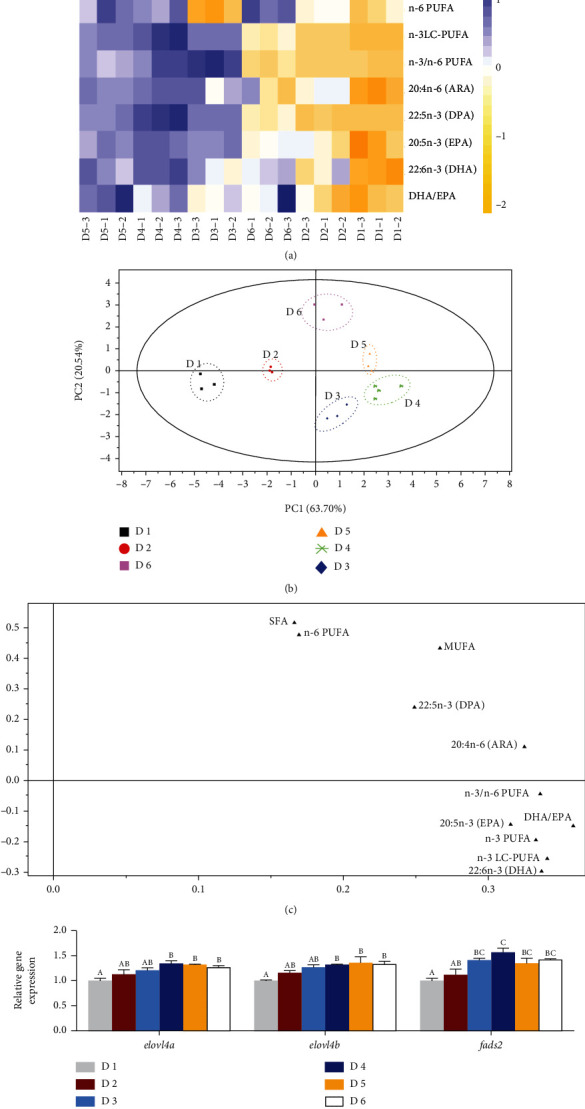
Effects of dietary lipid levels, fatty acid profiles, and LC-PUFA biosynthesis in the liver of juvenile *Acanthopagrus schlegelii* reared at low-salinity water. Heat map visualization of hepatic fatty acid composition (a), principal component analysis score plot (b), loading plot (c), and the expression levels of genes involved in LC-PUFA biosynthetic pathway (d). The expression levels of target gene were calculated relative to the expression level of *β-actin* and then presented as relative to the expression level in the D1 group. Values are means (*n* = 3), with standard errors represented by vertical bars. Mean values for the same column with different superscript letters were significantly different (*P* < 0.05). ARA: 20:4n-6; DHA: 22:6n-3; DHA/EPA: 22:6n-3/20:5n-3; *elovl4a*: elongase of very long chain fatty acids 4a; *elovl4b*: elongase of very long chain fatty acids 4b; EPA: 20:5n-3; *fads2*: fatty acyl desaturase 2; LC-PUFA: long-chain polyunsaturated fatty acid; MUFA: monounsaturated fatty acids; PUFA: polyunsaturated fatty acids; SFA: saturated fatty acids.

The effects of dietary lipid levels on the fatty acid profile of the liver in juvenile *A. schlegelii* reared at low-salinity water are presented in Table 5 and Figure 3. The contents of total saturated fatty acids (SFA), monounsaturated fatty acid (MUFA), arachidonic acid (20:4n-6, ARA), and docosapentaenoic acid (22:5n-3, DPA) in the liver increased significantly with increasing dietary lipid levels (*P* < 0.05). Meanwhile, the levels in the liver of n-3 PUFA, n-3 LC-PUFA, docosahexaenoic acid (22:6n-3, DHA), eicosapentaenoic acid (20:5n-3, EPA), and DHA/EPA all increased with increasing dietary lipid levels to 188.9 g/kg (D4) and then decreased after that (Table 5). Heat map visualization was conducted to indicate the macroscopic effects of dietary lipid levels on fatty acid composition of the liver (Figure 3(a)). All data were normalized, with blue color representing higher values and yellow color representing lower values. Results clearly showed that higher values of n-3 PUFA, n-3 LC-PUFA, n-3/n-6 PUFA, ARA, EPA, DHA, and DHA/EPA were recorded in fish fed with the diets supplemented with 188.9 g/kg and 239.3 g/kg lipid levels (D4 and D5 groups), and these two groups clustered close to each other. Principal component analysis (PCA) score plot and loading plot of fatty acid composition of the liver are presented in Figures 3(b) and 3(c), respectively. The first two principal components (PCs) were 84.24% of the variation (63.70% and 20.54% of the total variance, respectively). As shown in Figure 3(e), all replicates of these groups were divided into six clusters (cluster 1 (D1), cluster 2 (D2), cluster 3 (D3), cluster 4 (D4), cluster 5 (D5), and cluster 6 (D6)), and six clusters were intuitively separated. The PCA loading plot indicated the distribution of fatty acid data of the liver affected by dietary lipid levels. Combining the information in Figures 3(d) and 3(e), n-3 PUFA, n-3 LC-PUFA, n-3/n-6 PUFA, EPA, DHA, and DHA/EPA were at the under right of PC1, which were highly correlated with D3 and D4 groups.

The key gene expression levels of LC-PUFA biosynthetic pathway in the liver were significantly affected by the increase of dietary lipid levels (Figure 3(d)). The highest expression levels of elongase 4a (*elovl4a*), elongase 4b (*elovl4b*), and fatty acid desaturase 2 (*fads2*) increased up to 188.9 g/kg dietary lipid supplementation (D4) and significantly than the D1 group (*P* < 0.05). Similar result was found in elongase 4b (*elovl4b*) expression level; the highest value was recorded in the D5 group and significantly higher than fish fed with the diet supplemented with 68.7 g/kg lipid (*P* < 0.05) but showed no markedly difference with other treatments (*P* > 0.05).

### 3.4. Serum Biochemical Parameters, Biometric Indices, and Hepatic Histological Assay

**Figure 4:**
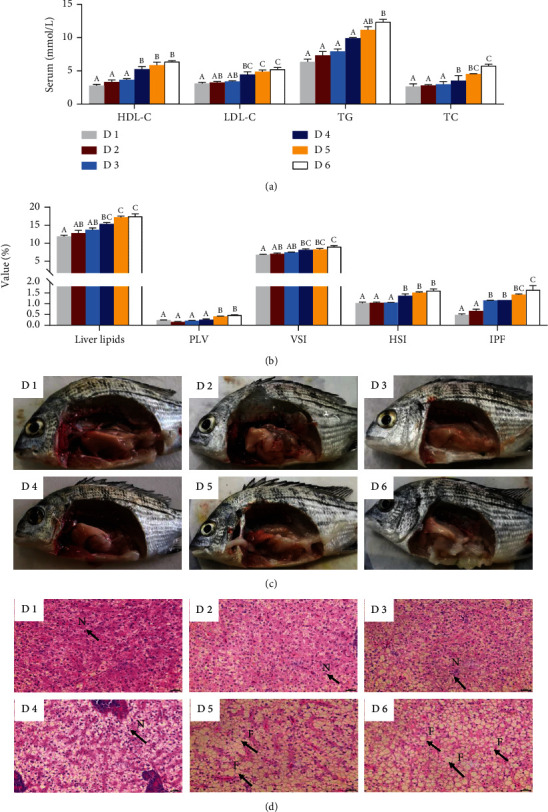
Effects of dietary lipid levels on serum biochemical indices and lipid accumulation of juvenile *Acanthopagrus schlegelii* reared at low-salinity water. Serum biochemical indices (a), lipid contents in the liver, PLV in paraffin section of the liver, and morphology index (b), mesenteric fat (c), and paraffin section of the liver with hematoxylin and eosin staining (×400, bar = 20 *μ*m) (d). Values are means (*n* = 3), with standard errors represented by vertical bars. Mean values for the same column with different superscript letters were significantly different (*P* < 0.05). F: lipid droplet vacuole; HDL-C: high-density lipoprotein cholesterol; HIS: hepatosomatic index; IPF: intraperitoneal fat ratio; LDL-C: low-density lipoprotein cholesterol; N: nucleus; PLV: percentage of lipid vacuoles; TG: triacylglycerol; TC: total cholesterol; VSI: viscerosomatic index.

Serum biochemical analysis showed that the contents of HDL-C, LDL-C, TG, and TC were significantly increased with the increase of dietary lipid levels under low-salinity environment (*P* < 0.05) (Figure 4(a)). Same patterns were found in PLV in histological section and morphological indices including VSI, HSI, and IPF as well as hepatic lipid content; those were significantly increased with the increase of dietary lipid levels (*P* < 0.05) (Figure 4(b)). Accordingly, the accumulation of intraperitoneal fat was increased with the increase of dietary lipid levels, especially in fish fed with the diets supplemented with 239.3 g/kg and 269.4 g/kg lipid (D5 and D6 groups) (Figure 4(c)). The results of hepatic paraffin section are presented in Figure 4(d). The hepatocyte shape and structure were regular and normal in fish fed the diet supplementing less than 188.9 g/kg lipid levels, and the nucleus with nucleolus was spherical and basically in the middle. However, the results of hepatic paraffin section displayed that when fish fed the diets containing 239.3 g/kg and 269.4 g/kg, dietary lipid levels increased vacuolar fat drops and induced hepatic fat pathological changes (Figure 4(d)).

### 3.5. Key Markers of Lipid Metabolism

**Figure 5:**
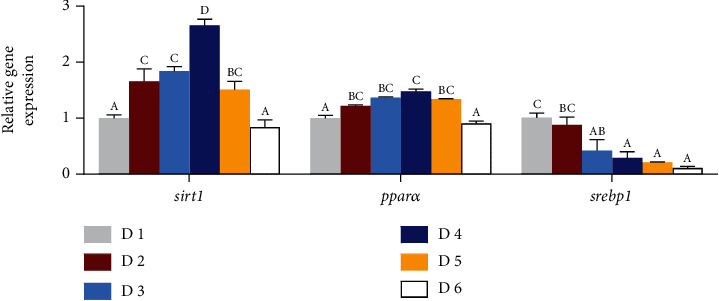
Effects of dietary lipid levels on lipid metabolism of juvenile *Acanthopagrus schlegelii* reared at low-salinity water. Values are means (*n* = 3), with standard errors represented by vertical bars. Mean values for the same column with different superscript letters were significantly different (*P* < 0.05). *pparα*: peroxisome proliferators-activated receptor alpha; *sirt1*: silent regulator 1; *srebp-1*: sterol regulatory element-binding protein-1.

The key gene expression levels of lipid metabolism in the liver were significantly affected by dietary lipid levels in juvenile *A. schlegelii* reared at low-salinity water (Figure 5). The relative expression level of sterol regulatory element-binding protein 1 (*srebp-1*) decreased significantly with the increase of dietary lipid levels, while the expression levels of silent information regulator 1 (*sirt1*) and peroxisome proliferator activated receptors alpha (*pparα*) firstly increased significantly with dietary lipid levels up to 188.9 g/kg (D4) and then markedly decreased with dietary lipid levels increasing (*P* < 0.05).

### 3.6. Oxidation and Antioxidant Index Assay

**Figure 6:**
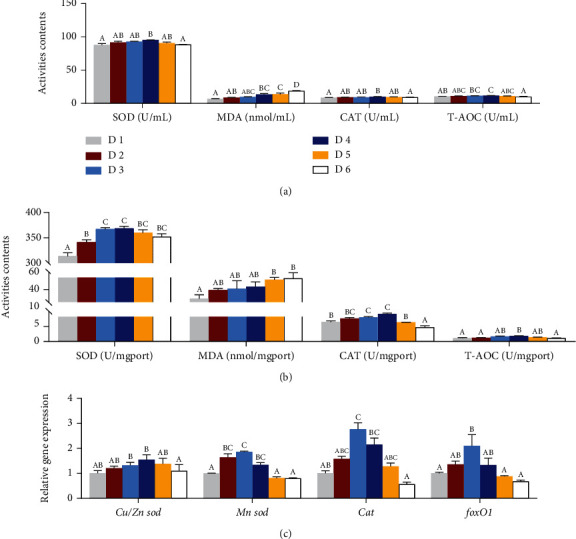
Effects of dietary lipid levels on oxidative stress and antioxidant capacity of juvenile *Acanthopagrus schlegelii* reared at low-salinity water. Oxidation and antioxidant parameters in serum (a) and liver (b) and the expression levels of genes involved in antioxidation in liver (c). Values are means (*n* = 3), with standard errors represented by vertical bars. Mean values for the same column with different superscript letters were significantly different (*P* < 0.05). CAT: catalase; *Cu-Zn sod*: Cu-Zn superoxide dismutase; MDA: malonaldehyde; *Mn sod*: Mn superoxide dismutase; T-AOC: total antioxidant capacity.

The oxidation and antioxidant parameters in the liver and serum were significantly affected by dietary lipid levels in juvenile *A. schlegelii* under low-salinity environment (Figure 6). The activities of SOD, CAT, and T-AOC in serum and liver increased significantly as dietary lipid levels increased from 68.7 g/kg up to 188.9 g/kg and then decreased with dietary lipid levels increasing (*P* < 0.05) (Figures 6(a) and 6(b)). However, the contents of MDA in serum and liver increased significantly with the increase of dietary lipid levels (*P* < 0.05) (Figures 6(a) and 6(b)). With regard to genes related to antioxidation in the liver, the expression level of Cu/Zn superoxide dismutase (*Cu/Zn sod*) in fish fed with the diet supplemented with 188.9 g/kg lipid was significantly higher than the D6 group (*P* < 0.05). Mn superoxide dismutase (*Mn sod*), catalase (*cat*), and forkhead box 1 (*foxO1*) increased significantly as dietary lipid levels from 68.7 g/kg up to 143.5 g/kg and then decreased with the increase of dietary lipid levels, but D2 and D4 treatments did not show any significant differences (*P* > 0.05) (Figure 6(c)).

### 3.7. Key Makers of ERS, Inflammation, and Cell Apoptosis in the Liver

**Figure 7:**
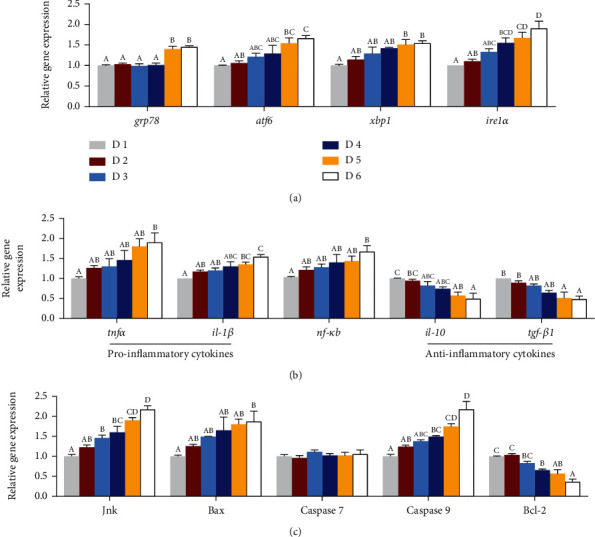
Effects of dietary lipid levels on the related gene expressions of endoplasmic reticulum stress (a), inflammatory responses (b), and apoptosis (c) in the liver of juvenile *Acanthopagrus schlegelii* reared at low-salinity water. Values are means (*n* = 3), with standard errors represented by vertical bars. Mean values for the same column with different superscript letters were significantly different (*P* < 0.05). *atf6*: activating transcription factor 6; *grp78*: glucose regulated protein 78; *ire1ɑ*: inositol requiring enzyme-1ɑ; *xbp1*: X-box binding protein 1; *il-10*: interleukin-10; *il-1β*: interleukin-1*β*; *nf-κb*: nuclear factor kappa B; *tgfβ*: transforming growth factor *β*; *tnfα*: tumor necrosis factor *α*; *bax*: bcl2-associated X; *bcl-2*: B cell leukemia 2; *jnk*: c-Jun N-terminal kinase.

Dietary lipid levels significantly affected the key gene expression levels of ERS (endoplasmic reticulum stress), inflammation, and cell apoptosis in the liver of *A. schlegelii* reared at low-salinity water (Figure 7). Dietary lipid levels increased from 239.3 to 269.4 g/kg significantly upregulated the expression levels of glucose-regulated protein 78 (*grp78*), activating transcription factor 6 (*atf6*), X-box binding protein 1 (*xbp1*), and inositol requiring enzyme-1 (*ire1α*) and were significantly higher than those in fish fed with the diet containing 68.7 g/kg lipid (D1) (*P* < 0.05) (Figure 7(a)). With regard to genes related to inflammation, fish fed with the high level of dietary lipid level (269.4 g/kg) showed markedly higher expression levels of interleukin-1 beta (*il-1β*), tumor necrosis factor alpha (*tnfα*), and nuclear factor kappa b (*nf-κb*) compared to the D1 group (*P* < 0.05), with an opposite trend, downregulation with increasing dietary lipid levels, observed for anti-inflammatory cytokine including transforming growth factor beta 1 (*tgfβ-1*) and interleukin-10 (*il-10*) (*P* < 0.05) (Figure 7(b)). Dietary lipid levels had a clear impact on the genes related to apoptosis, with c-Jun N-terminal kinase (*jnk*), bcl2-associated X (*bax*), and autophagy relative gene 9 (*caspase 9*) showing graded increased expression with increasing dietary lipid levels; opposite results were found in B cell leukemia 2 (*bcl-2*) (*P* < 0.05) (Figure 7(c)). Additionally, the expression levels of *caspase 7* did not show any statistical differences among treatments (*P* > 0.05) (Figure 7(c)).

## 4. Discussion

As a supplier of energy substances and essential fatty acids, lipid can be effectively decomposed for energy and provide fatty acids for tissue renewal to meet the need of physical growth and energy metabolism [35, 36]. In the present study, growth performance was significantly affected by dietary lipid levels under low-salinity environment. The values of WG, SGR, and FBW increased with dietary lipid levels increased from 68.7 g/kg to 188.9 g/kg and then decreased with further increases in the dietary lipid level. Analyzing the relationship of dietary lipid levels and weight gain by linear broken-line model indicated that the optimal dietary lipid requirement of juvenile *A. schlegelii* reared at low-salinity water is 196.0 g/kg. A previous study reported that juvenile *A. schlegelii* could adapt dietary lipid levels of 82.9~136.0 g/kg under normal-salinity environment [19, 20]. Obviously, these results were lower than that in the present study, which might be attributed to more energy are needed to maintain the stability of osmoregulation and more LC-PUFAs are required to increase the permeability of cell membrane under low-salinity environment. Similar result was also found in *Litopenaeus vannamei* that 60 g/kg dietary lipid level was sufficient for practical diets at normal water salinity (30 psu), while the 80 g/kg dietary lipid level met the requirement of growth at low-salinity water (2 psu) [21]. In addition, the present study demonstrated that fish fed excessive dietary lipid with the level of 239.3 g/kg or beyond could reduce growth performance, which has been supported by previous studies in other fish species [37, 38]. Overall, the optimal dietary lipid requirement for juvenile black seabream reared at low-salinity water is 196.0 g/kg.

When salinity changes within a certain range, all euryhaline fish species can maintain a relatively stable internal environment for their osmoregulation ability [39]. The mechanisms of maintaining osmoregulation in fish involve various physiological responses and metabolic regulation, including maintaining osmosis/ion balance through changes in enzyme activity, hormone level, and ion concentration [39, 40]. In aquatic animals, the gill, kidney, and intestine are key tissues to cope with water salinity changes [41–43]. Cortisol plays an important role in osmotic regulation, which is involved in regulating ion absorption in many teleost fishes [44, 45]. Besides, NKA is a membrane protein that couples the active exchange of two extracellular K^+^ ions for three intracellular Na^+^ ions to the hydrolysis of one molecule of ATP, maintaining the K^+^ and Na^+^ concentration gradients between the cytosol and the extracellular fluid [46, 47]. In this study, the cortisol, Na^+^, and K^+^ concentrations in fish fed with the diet supplemented with moderate lipid levels (143.5 g/kg and 188.9 g/kg) were significantly higher than those in the D1 group, while an opposite result was found in Cl^−^ content. Accordingly, NKA activity in the gills and intestine increased with the increase of dietary lipid levels while showed a peak in the D4 group (188.9 g/kg) before declining with further lipid levels. These results indicated that supplemented appropriate dietary lipid level (188.9 g/kg) in diet could improve NKA activity in the gill and intestine to accelerate ion transport rate to adapt low-salinity environment. Previous studies reported that *ostf1* and *ncc* were upregulated at both mRNA and protein levels challenged by salinity changes in low permeability [48, 49]; otherwise, *nkaα* can be improved by the exchange of membrane Na^+^ and K^+^ in gill epithelial cells of euryhaline fishes [25, 50]. Besides, *aqp1* plays an important role in maintaining ionic and osmotic balance [51]. Hence, these key gene expression levels were measured herein, and the expression levels of *ostf1*, *ncc*, *aqp1*, and *nkaα* in both the gill and intestine were all significantly increased firstly and then decreased with dietary lipid levels increasing, peaking at D3, D4, or D5 groups (143.5 g/kg~239.3 g/kg), respectively, except for *nkaα* in the intestine. These results indicated that fish fed the diets supplemented with 143.5 g/kg~239.3 g/kg lipid could improve the osmoregulatory ability of *A. schlegelii* by activating the key gene expression levels of osmoregulation.

Fatty acids, especially n-3 LC-PUFAs, play critical roles in increasing the unsaturation of cell membranes to maintain normal physiological function and ion transport [52]. The present study showed that the levels of n-3 PUFA, n-3 LC-PUFA, DHA, EPA, and DHA/EPA in the liver were significantly increased by dietary lipid level of 188.9 g/kg (D4 group). The heat map clearly indicated that fish fed with moderate levels of dietary lipid (D3, D4, and D5 groups) clustered together. Furthermore, PCA scatter plot and score plot of hepatic fatty acid composition showed that DHA, EPA, n-3 PUFA, and n-3 LC-PUFA were at the lower right of the score plot and highly correlated with the D4 group. These results indicated that optimal dietary lipid supplementation (188.9 g/kg) could accelerate accumulation of n-3 PUFA, especially n-3 LC-PUFA in the liver, and, hence, improve adaptability to low-salinity environment. Moreover, evidence showed that appropriate dietary lipid supplementation could upregulate the gene expression levels related to LC-PUFA biosynthetic pathway including *elove4a*, *elovl4b*, and *fads2* in *L. vannamei*, thereby promoting the accumulation of LC-PUFA of the liver in response to adaptation to low-salinity water [41]. Indeed, similar results were found in the present study that fish fed the diet containing 188.9 g/kg lipid had significantly higher expression levels of *elovl4a*, *elovl4b*, and *fads2* compared to the D1 group. Hence, the present study showed that dietary lipid supplementation of 188.9 g/kg could increase accumulation of n-3 LC-PUFAs via activating key gene expression levels of LC-PUFA biosynthetic pathway in *A. schlegelii* and, consequently, maintain cell membrane permeability and ion balance, which has also been confirmed in a previous study for *Penaeus vannamei* [53]. However, further studies are needed in the future.

Evidence showed that excessive lipid intake could disrupt lipid homeostasis and result in lipid accumulation, which have been reported in various marine fish species reared at normal water salinity, such as *Pseudosciaena crocea* R. [37], Scophthalmus maximus L. [54, 55], and *A. schlegelii* [28, 56, 57]. In the present study, serum TG, TC, and LDL-C concentrations increased significantly with the increase of dietary lipid levels, indicating that excessive lipid intake could lead to lipid accumulation in blood system, which have also supported by previous studies that the concentrations of TC and TG are positively correlated with the dietary lipid levels [58–61]. Morphological indexes VSI, HSI, and IPF as well as lipid content in the liver increased with the increase of dietary lipid levels. Accordingly, photographs of intraperitoneal fat and paraffin section also confirmed that lipid accumulation and lipid drops could be caused when *A. schlegelii* reared at low-water salinity and fed dietary lipid levels of 239.3 g/kg or beyond. Hence, the current study revealed that supplemented dietary lipid levels of 239.3 g/kg or beyond could lead to lipid accumulation and lead to liver damage in juvenile *A. schlegelii* reared at low-water salinity. Moreover, lipid metabolism includes many complex biochemical processes such as lipid absorption, anabolism, and catabolism, which are regulated by some key genes and transcription factors [62, 63]. Sirt1 is considered to be the master switch regulating energy homeostasis, and evidence has shown that *sirt1* has a negative regulation effect on *srebp-1*, but with a positive regulation effect on *pparα* [64–66]. Generally, lipid metabolism is regulated by dietary lipid levels; in this study, results showed that the expression levels of *sirt1* and *pparα* were markedly upregulated by dietary lipid levels of 188.9 g/kg (D4 group) when compared to D1 and D6 groups, whereas the expression level of *srebp-1* decreased significantly with the increase of dietary lipid levels. These results indicated that appropriate dietary lipid level (188.9 g/kg) could promote lipolysis and suppress lipogenesis of *A. schlegelii* reared at low-salinity water.

In the process of evolution, organisms have generated many defense systems as protective mechanisms to prevent oxidative stress [67, 68]. Specifically, SOD is the first line of defense in the antioxidant defense system, which is responsible for removing O^2−^, Cu/Zn SOD, and Mn SOD which are the most widely distributed metal binding enzymes in the extracellular and matrix of various organisms [69, 70]. CAT is one of the key antioxidant enzymes, which can catalyze the conversion of two H_2_O_2_ into water and oxygen molecules [71], while MDA is a major metabolic marker of oxidative damage [72]. In the present study, the activities of SOD, CAT, and T-AOC in serum and liver were increased with the dietary lipid levels increased from 68.7 g/kg to 188.9 g/kg and then decreased with further increase of lipid levels. Nevertheless, MDA content in serum and liver increased significantly with the increase of dietary lipid levels, and fish fed with the diet containing 239.3 g/kg and 269.4 g/kg lipid (D5 and D6) was significantly higher than the D1 group. These findings showed that excessive lipid intake (239.3 g/kg and 269.4 g/kg) could cause OS by generating high values of MDA in tissues of *A. schlegelii* reared at low-salinity water, while optimal dietary lipid level of 188.9 g/kg (D4) could reduce OS by improving antioxidant capacity; these results have also been confirmed in various fish species including *S. maximus* L., *Takifugu rubripes*, and *Megalobrama amblycephala* that high lipid intake could increase tissue MDA and decrease antioxidant capacity [54, 73, 74]. Furthermore, the relative expression levels of antioxidant genes *Cu/Zn sod*, *Mn sod*, *cat*, and *foxO1* were also measured herein. Results showed that fish fed the diet supplemented with moderate lipid levels of 143.5~188.9 g/kg (D3 and D4 groups) dramatically upregulated the gene expression levels of *Cu/Zn sod*, *Mn sod*, *cat*, and *foxO1*. In conclusion, when *A. schlegelii* reared at low-salinity water, the appropriate dietary lipid level (143.5-188.9 g/kg) can improve antioxidant capacity and eliminate metabolic by-products (such as free radicals), thus alleviating OS caused by low salinity and lipid oxidation with the increase of dietary lipid levels.

Maintaining endoplasmic reticulum (ER) homeostasis is the basis for normal lipid metabolism [75]. ER stress (ERS) can be caused by lipid metabolism disorder, etc. [76, 77]. Under normal conditions, *ire1α* and *atf6* remain relatively stable by binding to *grp78* [78, 79], while when ERS occurs, *grp78* would be dissociated, thereby activating *ire1α* and *atf6* [80]. Evidence has shown that the activated *ire1α* could self-assemble into oligomeric complexes, thereby activating downstream *xbp1* expression [78, 81, 82]. In this study, the expression level of *grp78* was significantly upregulated by high dietary lipid levels of 239.3 g/kg and 269.4 g/kg (D5 and D6 groups). Accordingly, the expression levels of ERS-related genes *atf6*, *xbp1*, and *ire1α* were dramatically increased by dietary lipid levels of 239.3 g/kg and 269.4 g/kg (D5 and D6 groups). These results indicated that excessive dietary lipid intake (239.3 g/kg and 269.4 g/kg) could lead to ERS of *A. schlegelii* reared under low-salinity environment. Similar results were also found in various fish species such as *M. amblycephala*, *Oreochromis niloticus*, and *Larimichthys crocea* [59, 83, 84] In addition, a previous study suggested that NF-*κ*B might be involved in regulation of ERS [85]. Hu et al. [86] reported that IRE1 could mediate NF-*κ*B activation and, hence, lead to inflammation. Indeed, in the present study, the gene expression levels of *nf-κb* and proinflammatory cytokines (*il-1β* and *tnfα*) in the liver were significantly upregulated with dietary lipid levels increasing, and the highest expression levels were found in dietary lipid level of 269.4 g/kg (D6), which were positively regulated by *ire1α*. On the contrary, the expression levels of anti-inflammatory cytokines *tgf-β1* and *il-10* were significantly downregulated with the increase of dietary lipid levels, and those fish fed with the dietary lipid levels of 239.3 g/kg and 269.4 g/kg (D5 and D6 groups) were markedly lower than D1 and D2 groups. These results indicate that excessive dietary lipid intake (239.3 g/kg and 269.4 g/kg) could cause inflammation of *A. schlegelii* reared at low-salinity water, but those fed with optimal dietary lipid level (188.9 g/kg) could not trigger inflammatory response. Moreover, evidence demonstrated that IRE1*α* could also activate JNK [87, 88]. In the current study, the expression levels of *jnk* as well as the proapoptosis factors *bax* and *caspase 9* in the liver were significantly upregulated with the increase of dietary lipid levels; the highest expression levels were recorded in dietary lipid level of 269.4 g/kg (D6) and significantly higher than D1 group, while opposite result was found in the expression level of *bcl-2*. These findings showed that apoptosis would be caused when *A. schlegelii* reared at low-salinity water and fed with high dietary lipid level of 269.4 g/kg, which was consistent with a previously study in *M. amblycephala* that high fat diet could cause hepatocyte apoptosis [73].

## 5. Conclusion

**Figure 8:**
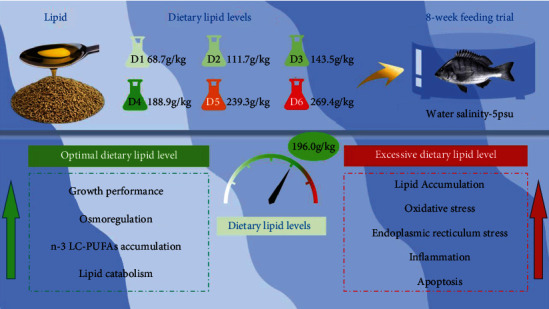
Effects of different dietary lipid levels on growth performance, osmoregulation, lipid metabolism, and physiological responses of juvenile *Acanthopagrus schlegelii* under low-salinity environment. Green/red arrows represent increase/upregulate.

In conclusion, based on weight gain, the optimal dietary lipid requirement of juvenile *A. schlegelii* is 196.0 g/kg by linear broken-line model analysis, which is quite close to the D4 group (188.9 g/kg). Optimal dietary lipid level could improve growth performance, osmoregulation ability, and antioxidant capacity of juvenile *A. schlegelii* reared at low-salinity water. Moreover, fish fed the diet containing lipid level of 188.9 g/kg increased n-3 LC-PUFA accumulation and, consequently, is beneficial to maintain osmotic pressure balance. Dietary lipid supplementation of 188.9 g/kg could maintain lipid homeostasis via upregulating the expression levels of *sirt1* and *pparα*. Nevertheless, fish fed the diet containing lipid levels of 188.9 g/kg or below could maintain ER relatively stable, but when the dietary lipid levels up to 239.3 g/kg or beyond, ERS could be caused, hence triggering inflammation and apoptosis by activated *nf-κb* and *jnk* expression. The findings of this study preliminarily clarify the lipid nutritional regulation strategy on improving the adaptability of *A. schlegelii* to low-salinity environment (Figure 8).

## Figures and Tables

**Table 1:** Ingredients and proximate composition of the experimental diets (g/kg, dry matter) [20].

Ingredients	Experimental diets					
	D1	D2	D3	D4	D5	D6
Fish meal	260.0	260.0	260.0	260.0	260.0	260.0
Soy protein concentrate	100.0	100.0	100.0	100.0	100.0	100.0
Soybean meal	160.0	160.0	160.0	160.0	160.0	160.0
Wheat flour	202.0	202.0	202.0	202.0	202.0	202.0
Fish oil	8.0	28.0	48.0	68.0	88.0	108.0
Soybean oil	8.0	28.0	48.0	68.0	88.0	108.0
Soybean lecithin	10.0	10.0	10.0	10.0	10.0	10.0
Vitamin premix^1^	10.0	10.0	10.0	10.0	10.0	10.0
Mineral premix^1^	20.0	20.0	20.0	20.0	20.0	20.0
Choline chloride	2.0	2.0	2.0	2.0	2.0	2.0
Ca(H_2_PO4)_2_	20.0	20.0	20.0	20.0	20.0	20.0
Cellulose	200.0	160.0	120.0	80.0	40.0	0.0
SUM	1000.0	1000.0	1000.0	1000.0	1000.0	1000.0
*Proximate composition*						
Dry matter	884.9	895.0	921.0	887.4	884.7	915.7
Crude protein	367.9	364.1	370.9	371.3	396.2	384.4
Crude lipid	68.7	111.7	143.5	188.9	239.3	269.4
Ash	103.1	102.5	117.7	105.6	108.4	107.7
Energy (MJ/kg)	14.06	15.68	17.09	18.90	21.48	22.39
Protein/energy (g protein/MJ)	26.16	23.23	21.70	19.65	18.45	17.17

^1^Vitamin premix and mineral mixture were supplied by Beijing Sunpu Biochemical Technology Co., Ltd. (Beijing China).

**Table 2:** Fatty acid composition (mg/g, dry matter) of the experimental diets.

Fatty acid	Experimental diets					
	D1	D2	D3	D4	D5	D6
14:0	2.21	2.83	3.64	4.30	5.38	6.92
16:0	11.84	14.88	18.71	21.67	27.59	34.73
18:0	2.87	4.00	5.35	6.41	8.39	11.04
20:0	0.19	0.30	0.44	0.56	0.72	0.98
∑SFA^1^	17.11	22.00	28.14	32.95	42.09	53.67
16:1n-7	1.98	2.64	3.43	3.93	5.23	6.82
18:1n-9	13.76	21.89	29.48	34.30	47.82	62.83
20:1n-9	0.42	0.60	0.81	0.97	1.35	1.80
22:1n-11	0.08	0.12	0.16	0.17	0.20	0.27
∑MUFA^2^	16.25	25.24	33.88	39.37	54.59	71.72
18:2n-6	6.75	9.69	11.72	12.28	19.02	25.63
18:3n-6	0.04	0.05	0.05	0.05	0.06	0.06
20:2n-6	0.07	0.10	0.17	0.20	0.24	0.33
20:4n-6 (ARA)	0.33	0.43	0.47	0.52	0.77	1.21
∑n-6 PUFA^3^	7.19	10.27	12.42	13.05	20.10	27.22
18:3n-3	1.79	2.71	3.27	3.68	5.75	8.31
18:4n-3	0.40	0.52	0.58	0.66	1.02	1.52
20:4n-3	0.15	0.23	0.26	0.39	0.47	0.70
20:5n-3 (EPA)	3.14	3.32	3.68	4.06	4.78	7.16
22:5n-3 (DPA)	0.45	0.51	0.53	0.63	0.82	1.30
22:6n-3 (DHA)	4.45	5.42	5.39	6.73	9.46	15.55
∑n-3 PUFA^4^	10.38	12.70	13.71	16.16	22.30	34.54
n-3/n-6 PUFA^5^	1.44	1.24	1.10	1.24	1.11	1.27
n-3 LC-PUFA^6^	8.04	9.24	9.60	11.42	15.07	24.01
DHA/EPA^7^	1.42	1.63	1.46	1.66	1.98	2.17

D1: 68.7 g/kg dietary lipid; D2: 111.7 g/kg dietary lipid; D3: 143.5 g/kg dietary lipid; D4: 188.9 g/kg dietary lipid; D5: 239.3 g/kg dietary lipid; D6: 269.4 g/kg. ^1^SFA: saturated fatty acids; ^2^MUFA: monounsaturated fatty acids; ^3^n-6 PUFA: n-6 polyunsaturated fatty acids; ^4^n-3 PUFA: n-3 polyunsaturated fatty acids; ^5^n-3/n-6 PUFA: the ratio of n-3 PUFA to n-6 PUFA; ^6^n-3 LC-PUFA: n-3 long-chain PUFA; ^7^DHA/EPA: the ratio of DHA to EPA.

**Table 3:** Real-time quantitative PCR primers for genes and *β-actin* of juvenile black seabream (*Acanthopagrus schlegelii*).

Gene	Nucleotide sequence (5′-3′)	Size (bp)	Accession no. or publication
*ostf1* ^1^	F: GTGGAAGTGCAGGTGTAGGA	243	OL321591
	R: CAGAGCCACAGGAGGAGAAA		
*ncc* ^2^	F: TTGCTGTTGTCGGCTCG	235	OL321595
	R: CCACGCTGGGTTTGTCC		
*aqp1* ^3^	F: CAGGATTAAAGTGTGCCCCG	151	OK338012
	R: TTGCTGTTCATATTCGCGGG		
*nkaα* ^4^	F: CCAGATCAGCGTGTTCAAGG	224	OL321586
	R: CATCACGCCGCCTTTTATCA		
*elovl4a* ^5^	F: CTACTCAGACGACCCCAA	164	KY348832
	R: CACCAGAGCGTGAACATG		
*elovl4b* ^6^	F: ATCCAGTTCCACGTGACCAT	222	KU372150
	R: TCCCATTTTCCTCCACCTCC		
*fads2* ^7^	F: AGCCAGGACCGAAATAAAA	113	KX058437
	R: AGGTGGAGGCAGAAGAACA		
	R: TTGCCATCCCACTTCTGC		
*sirt1* ^8^	F: TGGATGAAACTGTAGGAACC	238	MN871952
	R: ACAACAATGGACTGGGAA		
*ppar α* ^9^	F: ACGACGCTTTCCTCTTCCC	183	KX066234
	R: GCCTCCCCCTGGTTTATTC		
*srebp-1* ^10^	F: TGGGGGTAGGAGTGAGTAG	247	KX066235
	R: GTGAAGGGTCAGTGTTGGA		
*Cu/Zn sod* ^11^	F: CACGGTAAGAATCATGGCGG	202	OL321588
	R: TCTCCTCGTTGCCTCCTTTT		
*Mn sod* ^12^	F: TCTCTTTCTCGTAGCCCAGC	247	OL321589
	R: GCAAAGGGAGATGTGACAGC		
*cat* ^13^	F: CACGGTAAGAATCATGGCGG	237	OL321590
	R: TCTCCTCGTTGCCTCCTTTT		
*foxO1* ^14^	F: GAAGCTGTTCCACGCATCAA	150	OL752705
	R: TTCACCAAGAGCAGAGGGAG		
*grp78* ^15^	F: AACCAGCTGACCTCTAACCC	164	MT451934
	R: ATGTCTTCATCTGGCCACCA		
*atf6* ^16^	F: CCTGTTGGGTTTCTCCTCAG	222	MT512507
	R: CCGTTACTTCACAGTCAATCTGC		
*xbp1* ^17^	F: TGATATCGGGGAAGCAGACC	235	MW589390
	R: TTCCTGTCTCTGGCTGTCTG		
*ire1α* ^18^	F: AGAGGTCTTGGGTCATGGTG	181	OL361769
	R: GTCCCTCTCAGTGCAGAAGT		
*nf-κb* ^19^	F: AGCCCAAGGCACTCTAGACA	154	MK922543
	R: GTTCTGGGCAGCTGTAGAGG		
*tnfα* ^20^	F: GGAGACAGACGAGGGCAAGA	196	AY335443
	R: TCAGCCGCAAGCGTTATCTC		
*tgf-β1* ^21^	F: GGGTTTCCAACTTCGGC	209	[32]
	R: TTGTGTCCGTGGAGCGT		
*il-10* ^22^	F: TGTCAAACGGTTCCTTGCAG	172	MK922542
	R: GGCATCCTGGGCTTCTATCT		
*il-1β* ^23^	F: CATCTGGAGGCGGTGAA	231	JQ973887
	R: CGGTTTTGGTGGGAGGA		
*jnk* ^24^	F: ATAGCGTGTGGTCTGGGAAA	171	OK315340
	R: CGCAGACATGTAAACAGCCA		
*caspase 7*	F: GTTTGCCTACTCCACTGTGC	152	OL321593
	R: TGGCCACCATGTAGTTGACT		
*caspase 9*	F: CCATTGTTTCTGCAGTGCCT	214	OL321594
	R: GAGTAGTACTGGGTCTGGGC		
*bcl-2* ^25^	F: GCTCCAACGACTGATCAACC	203	OL420679
	R: TGACCTGAAGAACCCAGCTT		
*bax* ^26^	F: AAGTGGATGGACAGAGTGGG	232	OL321596
	R: ATGCAATCTGGTGGTGGAGA		
*β-Actin*	F: ACCCAGATCATGTTCGAGACC	212	[33]
	R: ATGAGGTAGTCTGTGAGGTCG		

^1^ *ostf1*: osmotic stress transcription factor 1; ^2^*ncc*: Na^+/^Cl^−^ cotransporter; ^3^*aqp1*: aquaporin; ^4^*nkaα*: Na^+^/K^+^-ATPase; ^5^*elovl4a*: elongase 4a; ^6^*elovl4b*: elongase 4b; ^7^*fads2*: fatty acid desaturase 2; ^8^*sirt1*: silent regulator 1; ^9^*pparα*: peroxisome proliferator activated receptors alpha; ^10^*srebp-1*: sterol regulatory element-binding protein 1; ^11^*Cu/Zn sod*: Cu/Zn superoxide dismutase; ^12^*Mn sod*: Mn superoxide dismutase; ^13^*cat*: catalase; ^14^*foxO1*: forkhead box 1; ^15^*grp78*: glucose regulated protein 78; ^16^*atf6*: activating transcription factor 6; ^17^*xbp1*: X-box binding protein 1; ^18^*ire1α*: inositol requiring enzyme-1; ^19^*nf-κb*: nuclear factor kappa b; ^20^*tnfα*: tumor necrosis factor alpha; ^21^*tgf-β1*: transforming growth factor *β*1; ^22^*il-10*: interleukin-10; ^23^*il-1β*: interleukin-1 beta; ^24^*jnk*: c-Jun N-terminal kinase; ^25^*bcl-2*: B cell leukemia2; ^26^*bax*: bcl 2-associated X.

**Table 4:** Growth performance, feed utilization, and survival rate of juvenile *Acanthopagrus schlegelii* fed experimental diets under low-salinity environment for 8 weeks.

Parameter	Experimental diets						ANOVA
	D1	D2	D3	D4	D5	D6	*P* value
IBW^1^ (g)	2.27 ± 0.00	2.26 ± 0.00	2.27 ± 0.01	2.27 ± 0.00	2.26 ± 0.00	2.27 ± 0.01	0.56
FBW^2^ (g)	12.88 ± 0.51^a^	15.32 ± 0.43^ab^	15.23 ± 0.28^ab^	19.76 ± 0.92^c^	17.12 ± 0.45^bc^	14.10 ± 0.65^a^	0.00
WG^3^ (%)	468.20 ± 22.27^a^	576.54 ± 19.53^ab^	581.29 ± 6.62^ab^	771.28 ± 41.64^b^	657.01 ± 19.06^bc^	522.05 ± 30.36^a^	0.00
SGR^4^ (%/d)	3.10 ± 0.07^a^	3.37 ± 0.03^abc^	3.43 ± 0.02^bc^	3.86 ± 0.09^d^	3.61 ± 0.04^cd^	3.26 ± 0.09^ab^	0.00
FE^5^	0.56 ± 0.06^a^	0.57 ± 0.05^a^	0.59 ± 0.01^a^	0.94 ± 0.11^b^	0.77 ± 0.05^ab^	0.70 ± 0.03^ab^	0.00
SR^6^ (%)	91.11 ± 1.11	93.33 ± 1.93	94.44 ± 1.11	94.45 ± 2.22	93.33 ± 3.33	91.11 ± 1.11	0.71

D1: 68.7 g/kg dietary lipid; D2: 111.7 g/kg dietary lipid; D3: 143.5 g/kg dietary lipid; D4: 188.9 g/kg dietary lipid; D5: 239.3 g/kg dietary lipid; D6: 269.4 g/kg dietary lipid. Data are reported as the mean and SEM (*n* = 3). Values in the same column with different superscripts are significantly different (*P* < 0.05). ^1^IBW: initial body weight; ^2^FBW: final body weight; ^3^WG: weight gain; ^4^SGR: specific growth rate; ^5^FE: feed efficiency; ^6^SR: survival rate.

**Table 5:** Effects of dietary lipid levels on fatty acid composition (mg/g, dry matter) in the liver of juvenile *Acanthopagrus schlegelii* reared at low-salinity water.

Fatty acid	Experimental diets							
	D1	D2	D3	D4	D5	D6	*F* value	*P* value
14:0	3.94 ± 0.12	4.14 ± 0.32	4.40 ± 0.29	4.64 ± 0.08	4.64 ± 0.16	4.43 ± 0.15	1.83	0.18
16:0	32.22 ± 0.07^a^	33.47 ± 0.75^ab^	34.74 ± 0.19^bc^	35.66 ± 0.38^cd^	37.33 ± 0.24^d^	41.75 ± 0.12^e^	82.89	0.00
18:0	20.04 ± 0.09^a^	22.51 ± 0.77^ab^	22.88 ± 0.06^bc^	24.74 ± 0.37^bc^	25.43 ± 0.27^c^	36.38 ± 1.04^d^	104.86	0.00
20:0	0.60 ± 0.07	0.71 ± 0.04	0.74 ± 0.02	1.35 ± 0.55	0.98 ± 0.04	1.13 ± 0.05	1.56	0.24
∑SFA^1^	56.80 ± 0.19^a^	60.82 ± 0.42^b^	62.75 ± 0.38^b^	66.39 ± 0.15^cd^	68.39 ± 0.59^cd^	83.69 ± 1.02^d^	298.20	0.00
16:1n-7	5.30 ± 0.14	5.41 ± 0.10	5.24 ± 0.04	5.61 ± 0.13	5.60 ± 0.24	5.74 ± 0.28	1.23	0.36
18:1n-9	64.29 ± 0.82^a^	67.39 ± 0.38^ab^	68.48 ± 0.65^bc^	70.81 ± 0.32^c^	70.88 ± 0.21^c^	74.38 ± 1.09^d^	28.06	0.00
20:1n-9	1.83 ± 0.02	1.83 ± 0.01	1.86 ± 0.02	1.84 ± 0.02	1.87 ± 0.02	1.84 ± 0.01	0.74	0.61
22:1n-11	0.80 ± 0.02	1.17 ± 0.31	0.84 ± 0.01	0.88 ± 0.01	1.32 ± 0.38	0.84 ± 0.01	1.16	0.39
∑MUFA^2^	72.22 ± 0.95^a^	75.79 ± 0.37^b^	76.42 ± 0.68^bc^	79.15 ± 0.43^c^	79.67 ± 0.23^c^	82.78 ± 1.06^d^	28.13	0.00
18:2n-6	11.43 ± 0.21^b^	10.73 ± 0.11^ab^	9.66 ± 0.13^a^	10.70 ± 0.33^ab^	11.23 ± 0.43^b^	11.07 ± 0.44^ab^	4.18	0.02
18:3n-6	2.95 ± 0.01	3.23 ± 0.18	3.46 ± 0.09	3.66 ± 0.30	3.19 ± 0.13	3.34 ± 0.17	2.01	0.15
20:2n-6	0.55 ± 0.07	0.86 ± 0.06	0.56 ± 0.18	0.82 ± 0.42	0.88 ± 0.35	1.40 ± 0.70	0.69	0.64
20:4n-6	2.20 ± 0.05^a^	2.69 ± 0.04^bc^	2.83 ± 0.09^bc^	2.95 ± 0.02^c^	2.89 ± 0.02^bc^	2.59 ± 0.14^b^	13.80	0.00
∑n-6 PUFA^3^	17.12 ± 0.17^ab^	17.51 ± 0.03^bc^	16.51 ± 0.18^a^	18.13 ± 0.14^cd^	18.18 ± 0.20^cd^	18.40 ± 0.08^d^	25.19	0.00
18:3n-3	0.96 ± 0.11	0.86 ± 0.02	0.93 ± 0.05	0.92 ± 0.09	0.93 ± 0.01	0.92 ± 0.01	0.25	0.93
18:4n-3	3.50 ± 0.40^a^	5.54 ± 0.40^b^	4.01 ± 0.17^ab^	3.59 ± 0.09^a^	4.03 ± 0.04^ab^	4.88 ± 0.64^ab^	4.92	0.01
20:4n-3	0.87 ± 0.05^a^	0.93 ± 0.02^a^	0.92 ± 0.05^a^	1.15 ± 0.03^b^	0.93 ± 0.01^ab^	0.92 ± 0.06^a^	5.89	0.01
20:5n-3 (EPA)	2.60 ± 0.15^a^	3.09 ± 0.08^b^	3.44 ± 0.03^cd^	3.54 ± 0.01^d^	3.42 ± 0.05^abc^	3.16 ± 0.02^bc^	22.01	0.00
22:5n-3 (DPA)	12.21 ± 0.01^a^	12.47 ± 0.03^a^	16.82 ± 0.03^c^	18.12 ± 0.32^d^	16.52 ± 0.04^c^	13.71 ± 0.18^b^	269.05	0.00
22:6n-3 (DHA)	1.21 ± 0.03^a^	1.54 ± 0.07^b^	1.61 ± 0.04^bc^	1.80 ± 0.02^bc^	1.71 ± 0.05^c^	1.63 ± 0.03^bc^	21.63	0.00
∑n-3 PUFA^4^	21.36 ± 0.48^a^	24.42 ± 0.40^b^	27.73 ± 0.14^c^	29.13 ± 0.51^c^	27.57 ± 0.04^c^	25.23 ± 0.58^b^	47.35	0.00
n-3/n-6 PUFA	1.09 ± 0.01^a^	1.11 ± 0.00^a^	1.46 ± 0.02^c^	1.41 ± 0.03^c^	1.32 ± 0.01^b^	1.13 ± 0.01^b^	111.18	0.00
n-3 LC-PUFA^5^	18.68 ± 0.06^a^	19.38 ± 0.02^a^	24.13 ± 0.05^c^	25.62 ± 0.50^d^	23.98 ± 0.05^c^	20.73 ± 0.22^b^	160.86	0.00
DHA/EPA	2.21 ± 0.05^a^	2.30 ± 0.05^ab^	2.41 ± 0.03^abc^	2.48 ± 0.03^bc^	2.57 ± 0.03^c^	2.51 ± 0.09^bc^	6.98	0.00

D1: 68.7 g/kg dietary lipid; D2: 111.7 g/kg dietary lipid; D3: 143.5 g/kg dietary lipid; D4: 188.9 g/kg dietary lipid; D5: 239.3 g/kg dietary lipid; D6: 269.4 g/kg. ^1^SFA: saturated fatty acids; ^2^MUFA: monounsaturated fatty acids; ^3^n-6 PUFA: n-6 polyunsaturated fatty acids; ^4^n-3 PUFA: n-3 polyunsaturated fatty acids; ^5^n-3 LC-PUFA: n-3 long-chain PUFA.

## Data Availability

The original contributions presented in the study are included in the article. Further inquiries can be directed to the corresponding author.
